# Safety and Efficacy of Low-Dose Corticosteroids in Patients With Non-severe Coronavirus Disease 2019: A Retrospective Cohort Study

**DOI:** 10.7759/cureus.12544

**Published:** 2021-01-07

**Authors:** Talal Almas, Maryam Ehtesham, Abdul Wali Khan, Tarek Khedro, Salman Hussain, Mehwish Kaneez, Reema Alsufyani, Dana Almubarak, Fatimah Alahmed, Hasan Alaeddin

**Affiliations:** 1 Internal Medicine, Royal College of Surgeons in Ireland, Dublin, IRL; 2 Internal Medicine, College of Physician and Surgeons Pakistan, Peshawar, PAK; 3 Internal Medicine, Hayatabad Medical Complex Peshawar, Peshawar, PAK; 4 Internal Medicine, Rawalpindi Medical University, Rawalpindi, PAK

**Keywords:** steroids, non-severe, covid-19

## Abstract

Background

To date, several pharmacological agents have been employed in the treatment and management of the coronavirus disease 2019 (COVID-19). While the utility of corticosteroids in severe COVID-19 infection is now widely touted, their efficacy in thwarting the progression of non-severe disease remains elusive.

Methods

A retrospective cohort study involving 25 patients with a confirmed diagnosis of non-severe COVID-19 infection was conducted. Subjects were assigned to either the steroid or the non-steroid group. A low-dose, short-course corticosteroid regimen was administered for seven days and the disease outcomes were recorded and compared among the two groups. The Kolmogorov-Smirnov test was employed to discern the data normality.

Results

In patients treated with low-dose, short-course steroids, the overall all-cause mortality was significantly lower compared with the non-steroid group (8.3% and 61.5%, respectively; p = 0.005). The prevalence of acute respiratory distress syndrome in the steroid group was significantly lower than that in the non-steroid group at the seven-day mark (16.7% and 84.6%, respectively; p = 0.002). Within the steroid group, the incidence of developing secondary complications was also markedly lower than that in the non-steroid group.

Conclusions

In patients afflicted with non-severe COVID-19, the employment of low-dose, short-course corticosteroids may confer a therapeutic advantage, significantly curtailing the mortality rate, the length of hospital stay, and the risk of developing secondary complications.

## Introduction

The coronavirus disease 2019 (COVID-19) continues to cause soaring morbidity and mortality worldwide [1]. Due to the paucity of data surrounding the efficacy of various drug classes in the management of COVID-19, a definitive treatment regimen remains elusive. Therefore, optimized supportive care is the cornerstone of current clinical management [2]. Nevertheless, corticosteroids have remained an integral part of the pharmacological management of patients afflicted with a severe COVID-19 infection in the aftermath of the RECOVERY trial [3]. The guidance issued by the World Health Organization recommends the use of systemic corticosteroids only in cases of severe COVID-19, advising against the uptake of steroids in mild and moderate infection [4]. However, the recommendations against the latter were based merely on the result of a single randomized control trial [3]. While various studies have demonstrated worse clinical outcomes secondary to the use of corticosteroids, the vast majority of the results have been linked to the administration of high-dose corticosteroids for a prolonged duration [5]. At present, there is a dearth of data evaluating the efficacy of a low-dose, short-course corticosteroid regimen in the treatment of a non-severe COVID-19 infection. Some studies have posited that the early administration of low-dose, short-course corticosteroids in mild and moderate COVID-19 cases has the potential to halt the progression of the disease process and improve clinical outcomes with no added adverse effects [6,7].

Corticosteroids boast immunosuppressive and anti-inflammatory properties that are mediated by the binding of glucocorticoids (GC) to GC receptors, thereby inhibiting the transcription of cytokines such as interleukin 6 (IL-6) that remain a hallmark of an aberrant immune response characteristic of a COVID-19 infection [8]. According to the current medical literature, steroids are effective in decreasing the overall mortality, the length of required oxygen therapy, and the mean length of hospital stay in patients infected with a severe COVID-19 infection [8]. Recent medical literature has further suggested that the short-term administration of low-dose methylprednisolone in the early stages of the disease can ameliorate the prognosis by thwarting the progression of a mild or moderate COVID-19 infection. Currently, there is anecdotal evidence regarding the uptake of steroids in the management of mild COVID-19 infections. Therefore, this retrospective cohort study aims to add to the existing literature by evaluating the early disease outcomes in patients with non-severe COVID-19 infection receiving a short course of low-dose corticosteroids in the initial stages of their infection.

## Materials and methods

The present retrospective cohort study involved 25 patients with a reverse transcription polymerase chain reaction (RT-PCR)-confirmed COVID-19 infection on a background history of clinical symptoms and radiological findings suggestive of non-severe COVID-19. A determination of the extent and severity of the disease process was made using the saturation values, and a persistent saturation level above 94% was deemed suggestive of a mild-to-moderate disease process. All patients included in the study were followed up until they recovered and were subsequently discharged. The patients included underwent a uniform therapeutic regimen, including azithromycin 500 mg once daily (OD), acetaminophen 500 mg BD, and cetirizine 10 mg OD. Furthermore, the patient comorbidities among the population were controlled using their routine pharmacological regimens, including antihypertensives. Within our patient population, 12 out of the 25 patients included received a short course of low-dose steroid therapy (prednisolone 5 mg QDS) for seven days while 13 patients did not receive any steroids.

Patients who had a positive RT-PCR result but remained asymptomatic were excluded from the study. The exclusion criteria acted to ensure that only patients with moderate degrees of infection, as suggested by their clinical symptoms, were included in the final analysis. Additionally, patients who were adhering to a long-term corticosteroid regimen post-transplant or as part of management of any autoimmune condition were excluded from our study. The data collated were analyzed using the Statistical Package for Social Sciences (SPSS) version 25.0 (IBM SPSS Statistics, Armonk, NY, USA). The various characteristics of the study population based on gender, symptomatology, laboratory investigations, treatment regimen, and outcome were then tabulated. The normality of the data was discerned using the Kolmogorov-Smirnov test. We employed the independent samples t-test and chi-square test to establish the definitive association of steroid use with an improvement in laboratory parameters and the eventual disease outcomes. A p-value of less than 0.05 was deemed statistically significant.

## Results

The current study included 25 patients with an RT-PCR-confirmed non-severe COVID-19 infection. Of the included patients, 80% were males while 20% were females. The mean age of the study participants was 56.39 ± 12.36 years. The symptomatology of the study population is tabulated in Table 1.

**Table 1:** Prevalence of various clinical symptoms among the study participants.

Symptomatology		Frequency	Percentage
Fever	Yes	21	84%
	No	4	16%
Fatigue	Yes	23	92%
	No	2	8%
Myalgia	Yes	12	48%
	No	13	52%
Dry cough	Yes	22	88%
	No	3	12%
Dyspnea	Yes	21	84%
	No	4	16%
Nausea	Yes	12	48%
	No	13	52%
Diarrhea	Yes	6	24%
	No	19	76%
Vomiting	Yes	4	16%
	No	21	84%
Anosmia	Yes	6	24%
	No	19	76%
Altered taste	Yes	16	64%
	No	9	36%
Sputum production	Yes	2	8%
	No	23	92%
Travel history to endemic areas	Yes	1	4%
	No	24	96%

**Figure 1:**
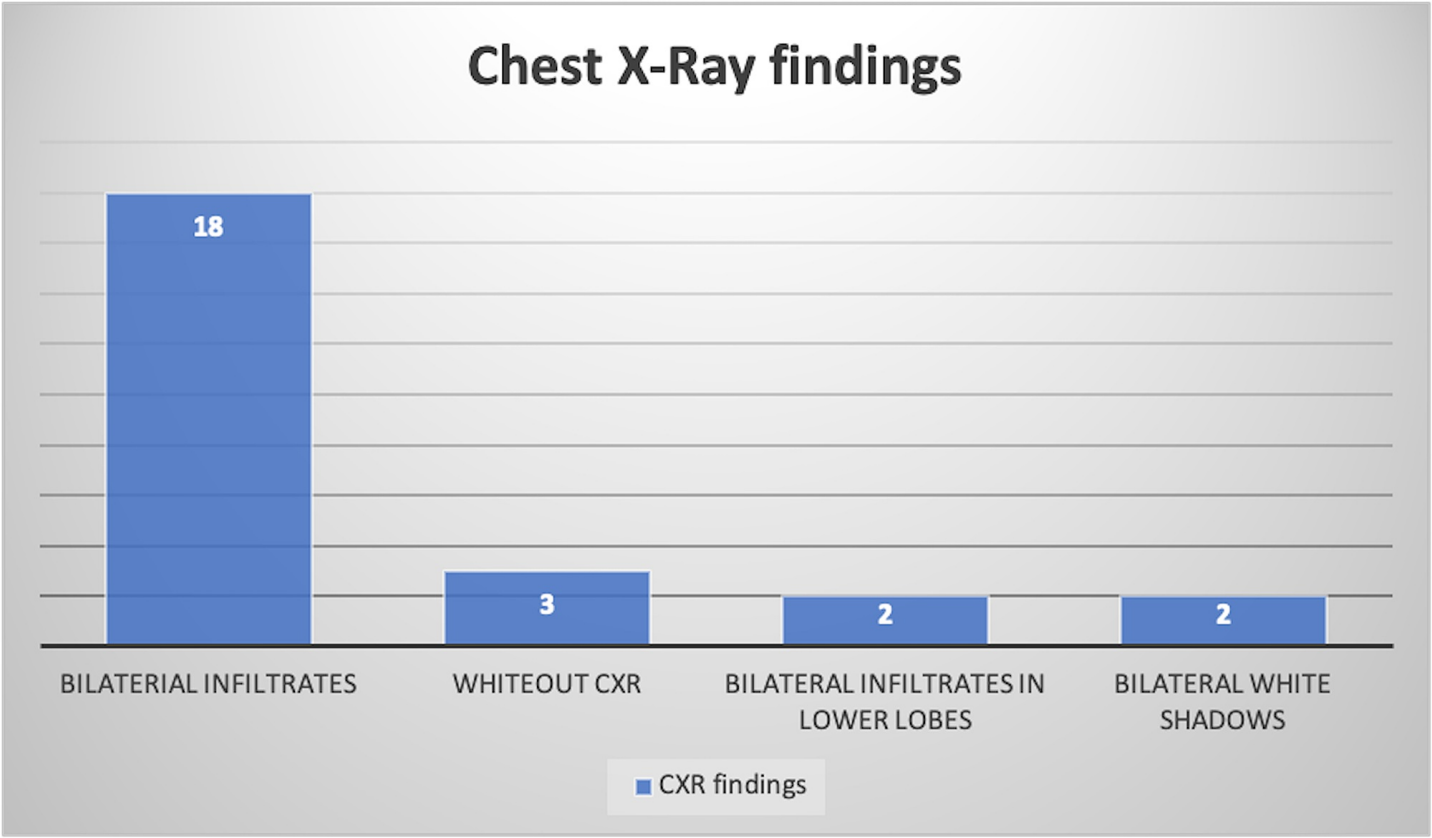
CXR findings observed among the study cohort. CXR: chest X-ray

Patients in our study population were further classified with respect to their chest X-ray (CXR) findings. The prevalence of the various CXR findings is elucidated in Figure 1.

**Figure 2:**
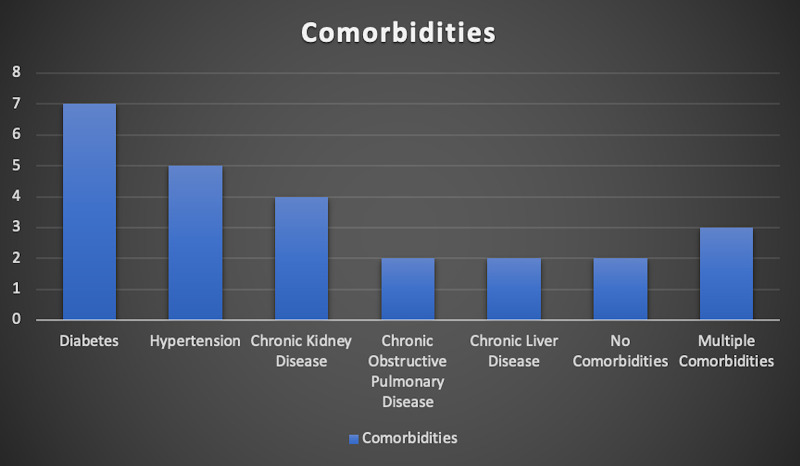
Baseline comorbidities of the study population.

Baseline comorbidities for the entire study population were established prior to the commencement of the study (Figure 2).

Patients within the study population were then assigned to either the steroid or the non-steroid group. Due consent was obtained from the patients prior to the commencement of the study. Both the groups were followed-up until they were discharged from the hospital or developed an adverse outcome secondary to the progression of their disease. The prevalence of these outcomes across the two groups is tabulated in Table 2.

**Table 2:** Comparison of the disease outcomes between the steroid and non-steroid groups. ARDS: acute respiratory distress syndrome *Chi-square test

Outcomes		Steroid group (n = 12)	Non-steroid group (n = 13)	p-value*
Survival status	Discharged/recovered	11	5	0.005
	Dead	1	8	
Oxygen requirement	Yes	9	12	0.521
	No	3	1	
Development of ARDS	Yes	2	11	0.002
	No	10	2	
Acute kidney injury	Yes	1	4	0.362
	No	11	9	
Acute cardiac injury	Yes	0	2	-
	No	12	11	
Shock	Yes	2	5	0.44
	No	10	8	
Secondary infection	Yes	1	4	0.362
	No	11	9	

**Figure 3:**
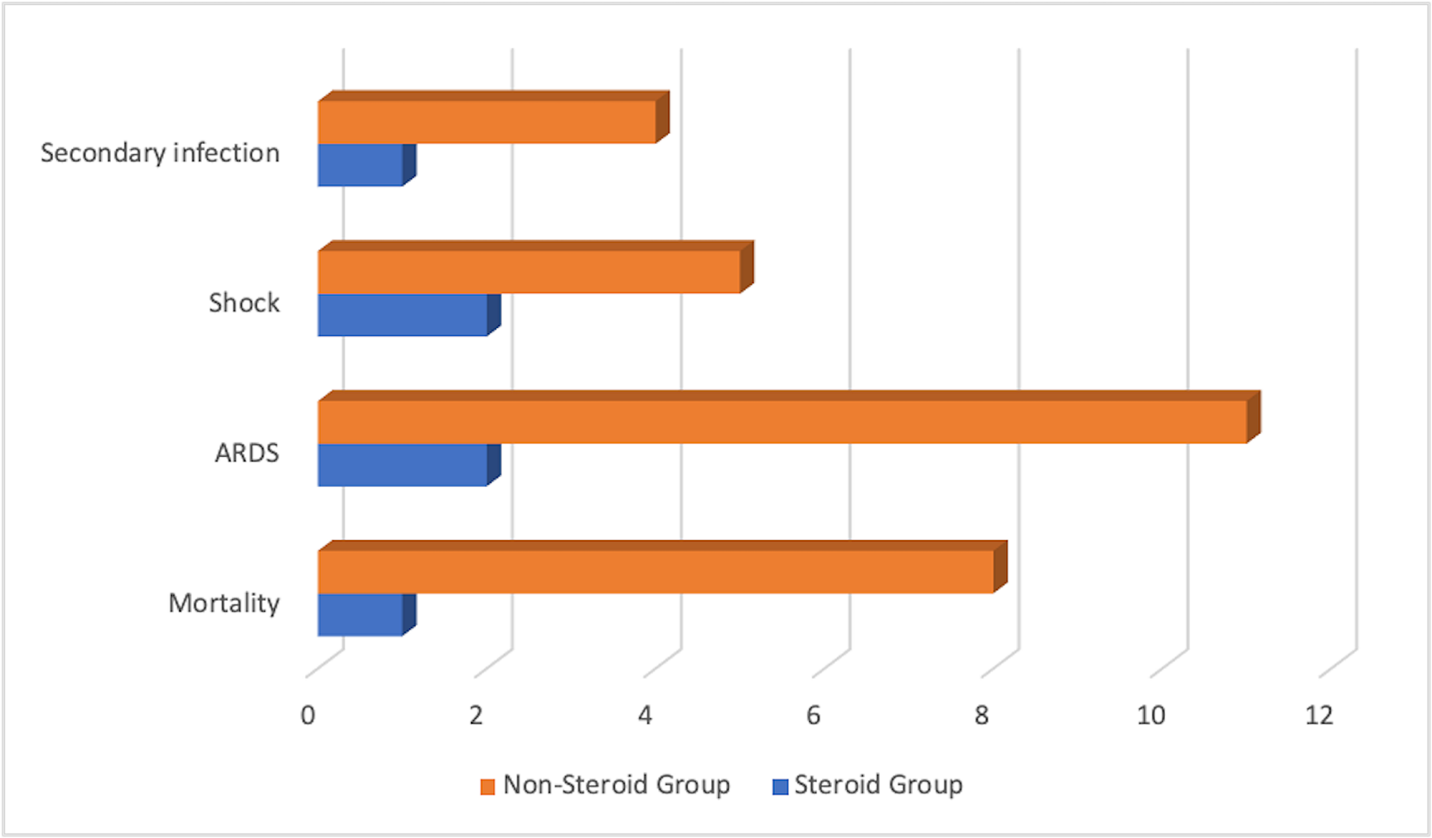
Comparison of the various adverse outcomes observed within the steroid and non-steroid groups. ARDS: acute respiratory distress syndrome

The mortality rate among the non-steroid group was 61.5% while the mortality rate in the steroid group was significantly lower at 8.3% (p = 0.005). Additionally, the prevalence of acute respiratory distress syndrome (ARDS) among the non-steroid group was 84.6% while the prevalence in the steroid group was 16.7% (p = 0.002). Furthermore, the risk of development of adverse outcomes such as acute kidney injury was also noted to be significantly higher among the non-steroid group compared with the steroid group. The comparison of these outcomes is delineated in Figure 3.

Various laboratory parameters that indicate the degree of inflammation and in turn the degree of disease severity were evaluated. These included the mean D-dimer levels, lactate dehydrogenase levels, and C-reactive protein (CRP) levels and were recorded on day seven of admission among both the groups. The results are delineated in Table 3.

**Table 3:** Comparison of various laboratory parameters among the steroid and non-steroid groups. LDH: lactate dehydrogenase; CRP: C-reactive protein; TLC: total lymphocyte count *Independent samples t-test

Parameters	Steroid group (n = 12)	Non-steroid group (n = 13)	p-value*
Mean D-dimer levels on day seven (ng/mL)	194.78 ± 54.75	293.91 ± 67.75	0.001
Mean LDH on day seven (U/L)	201.41 ± 33.75	242.19 ± 39.72	0.011
Mean CRP on day seven (mg/dL)	14.53 ± 5.17	44.43 ± 12.44	<0.001
Mean TLC (10^3^/µL)	8.9 ± 3.14	9.74 ± 3.76	0.552
Mean length of hospital stay (days)	14.23 ± 2.34	20.16 ± 3.46	<0.001

## Discussion

As the COVID-19 pandemic continues to wreak havoc, there has been mounting fervor surrounding the use of several pharmacological agents in the treatment of severe COVID-19 infections. Numerous pharmacological regimens have been investigated to date, including hydroxychloroquine, azithromycin, remdesivir, and tocilizumab [8]. More recently, corticosteroids have been one of the major focuses as part of potential life-saving therapy, and this guidance is predominantly derived from their use during the severe acute respiratory syndrome coronavirus 1 (SARS-CoV-1) and middle east respiratory syndrome coronavirus (MERS-CoV) outbreaks [8].

Over the past few months, numerous studies have examined the efficacy of corticosteroids. One cohort study showed that in COVID-19 and ARDS patients, methylprednisolone was associated with a lower risk of death [9]. Similarly, in Spain, methylprednisolone was shown to be associated with reduced mortality [10]. A study in Wuhan further showed that methylprednisolone reduced the duration of supplementary oxygenation by as much as six days in admitted patients [11]. Most notably, the RECOVERY trial showed that in patients hospitalized with severe COVID-19 disease, a 6 mg OD dose of dexamethasone for up to 10 days reduced the overall mortality compared to the non-steroid group (22.9% and 25.7%, respectively; p < 0.001) [12]. In general, the guidelines for treatment have vouched for the uptake of steroids only in severe disease forms and especially in intubated patients [13,14]. Nevertheless, there is paucity of data evaluating the use of low-dose corticosteroids administered for a short duration early in the disease course to thwart the progression of a mild or moderate COVID-19 infection [13]. In contrast, some studies have warned against the use of steroids in COVID-19 early in the disease course, citing their potential immunosuppressive properties as evidenced by their use in the previous pandemics [14]. In MERS-CoV, for instance, there was delayed viral clearance from the respiratory tract in patients adhering to a corticosteroid regimen [15]. Similarly, in SARS-CoV-1, hematologic viral RNA clearance was significantly lower in the steroid group, but the effect size was not quantified [16]. Therefore, the efficacy of corticosteroids in the management of non-severe COVID-19 infection remains enigmatic.

The current retrospective cohort study of 25 patients demonstrates that the use of a steroid regimen (prednisolone 5 mg QDS) was beneficial in the context of non-severe COVID-19 infection. Within the group receiving corticosteroids, the overall mortality was significantly lower, with the use of steroids portending a favorable prognosis in terms of the progression of mild and moderate COVID-19 infection. Similarly, significant differences in various laboratory parameters that correlate strongly with the severity of the disease process were noted. The CRP values in the steroid group were significantly lower at seven days when compared to the non-steroid group (14.53 mg/L and 44.43 mg/L, respectively; p < 0.001). Furthermore, there was a marked reduction in the length of hospital stay within the steroid group compared to the control or the non-steroid group (14.23 days and 20.16 days, respectively; p < 0.001).

Nevertheless, the implementation of a steroid regimen needs to be performed delicately. If used in excess or in immunocompromised patients, steroids can further downregulate the immune system, thereby impairing its ability to clear the viral load effectively [17]. These immunosuppressive effects of steroids can thus render a patient more susceptible to opportunistic or superimposed infections. This is especially concerning in the context of nosocomial pathogens [17]. Therefore, a low dose of corticosteroids for seven days might confer a therapeutic advantage by curbing the progression of non-severe disease while simultaneously avoiding unnecessary and potentially life-threatening immunosuppression. While the present study analyzed data obtained from merely 25 patients, its results can be extrapolated to shed light on the clinical value that low-dose, short-course steroids hold for thwarting the progression of non-severe COVID-19 infections.

## Conclusions

In patients hospitalized with a non-severe COVID-19 infection, the use of low-dose, short-course prednisolone for a duration of seven days effectively reduced the all-cause mortality and the total duration of hospital stay. Furthermore, the employment of the aforesaid steroids was associated with a significant amelioration of the patients’ clinical picture as evidenced by their significantly reduced levels of inflammatory markers.
